# An Insight into COPD Morphopathogenesis: Chronic Inflammation, Remodeling, and Antimicrobial Defense

**DOI:** 10.3390/medicina55080496

**Published:** 2019-08-17

**Authors:** Zane Vitenberga, Māra Pilmane, Aurika Babjoniševa

**Affiliations:** 1 Department of Morphology, Institute of Anatomy and Anthropology, Riga Stradins University, Kronvalda Boulevard 9, LV-1010 Riga, Latvia; 2 Pauls Stradins Clinical University Hospital, Pilsonu street 13, LV-1002 Riga, Latvia

**Keywords:** COPD, chronic inflammation, airways, tissue, immunohistochemistry

## Abstract

*Background and Objectives:* Intercellular signaling networks with high complexity cause a spectrum of mechanisms achieving chronic obstructive pulmonary disease (COPD) that still question many uncertainties. *Materials and Methods:* Immunoreactive cells in bronchial tissue obtained from 40 COPD patients and 49 healthy control subjects were detected by biotin-streptavidin immunohistochemistry method for the following markers of IL-1α, IL-4, IL-6, IL-7, IL-8, IL-10, IL-12, TNF-α, MMP-2, TIMP-2, TGF-β1, Hsp−70, hBD−2, hBD−3, hBD−4. *Results:* Overall the highest numbers (from mostly moderate (++) to abundance (++++)) of IL-1α, IL-4, IL-7, IL-8, IL-10, IL-12, MMP-2, TIMP-2, TGF-β1 immunoreactive cells were marked increasingly in the blood vessel wall, connective tissue, and bronchial epithelium of COPD-affected lung, respectively. We found statistically significant (*p* < 0.05) higher numbers of immunoreactive cells positive for all of examined interleukins, TNF-α, MMP-2, TIMP-2, TGF-β1, hBD-2, and hBD-3 in the COPD-affected lung compared to the control group, but not for Hsp-70 and hBD-4. *Conclusions:* COPD-affected lung tissue exhibits mostly inflammatory response patterns of increased IL-1α, IL-4, IL-8, IL-12, and TNF-α, especially in the airway epithelium. Increased MMP-2 and TGF-β1, but decreased Hsp-70, proposes pronounced tissue damage and remodeling in COPD. High numbers of hBD-2 and hBD-3 immunoreactive cells may highlight antimicrobial activity in COPD within stable regulation of local immunity.

## 1. Introduction

Chronic obstructive pulmonary disease (COPD) is a progressive disease determined by continuous airway obstruction, moreover, COPD is defined as a nationwide and worldwide health issue by being one of the leading causes of mortality and morbidity in respiratory and general medicine [1]. World Health Organization (WHO) data indicates COPD as being the third leading cause of death in the Top 10 causes worldwide already, in the year 2016. COPD prevalence worldwide is estimated to be around 251 million people [1,2].

Also named as risk factors, continuous exposure to cigarette smoke, exhaust fumes, and overall environmental pollution initiates cell and tissue damage, as well as oxidative stress by formation of free radicals. Although COPD is classically associated with the cigarette smoke exposition, at least 20% of COPD cases are associated with work place pollution like dust, chemical fumes, pesticides, steam, or other volatile and inhalable substances [3]. In some studies, nearly 70% of COPD patients are not smoking or never have [4]. Development of COPD is overall associated with the complex exposure to gaseous substances and their particles, individual internal factors (e.g., heredity, tissue local changes), persistent airway damage, dubious lung development, and maturation anamnesis, gender, social economic status, airway diseases [5,6].

Further on, these harms initiate cellular and tissue signalling pathways with increasing mediator release (like cytokines, remodelling factors) directly from cells being affected, e.g., bronchial epithelium, alveolar epithelium, alveolar macrophages [7], and fibroblasts [8]. Even the maximum elimination or risk factors still keeps the inflammation ongoing [9]. Overall, chronic inflammation, cell, and tissue damage remodelling processes in COPD are determined by molecular and cellular mechanisms [10]. Moreover, these events may point to the ontogenetic background and time-dependent microscopic environment of lung tissue, where the complex cell cross-talk and network of signalling molecules shape tissue architecture. The question is, to what continuing local changes and findings these events are leading [11]. The local environment of extensive molecular trafficking promotes inflammation, cell injury, and apoptosis determining the development of COPD [12]. Continuous chronic inflammation with immune cell infiltration, fibrosis, tissue remodelling, impairment of mucociliary clearance defence mechanisms, extensive mucus hypersecretion, and sequential emphysema highly dominates in COPD. Moreover, all events contribute to luminal narrowing of airway structures [13]. 

Intercellular signalling networks with high complexity, interrelations, and overall wide distribution of various mediators cause a spectrum of mechanisms achieving COPD. Inflammatory cytokines like interleukin (IL)-8 and tumour necrosis factor alpha (TNF-α) are highly expressed and released in airway tissue by various cells (e.g., bronchial epithelial cells, neutrophil leukocytes, tissue macrophages) orchestrating the inflammatory processes in COPD [14]. During the cell damage, released heat shock protein (Hsp)-70 further induces the release of IL-6 and IL-8 then holding cell and tissue damage and inflammatory processes alongside [15]. The COPD pathogenesis is highly associated with the local appearances and distribution of IL-1 (IL-1α, IL-1β) and IL-33 signalling pathways [16], IL-6, IL-8 [17], IL-4, IL-7 [18,19,20], IL-10 [21], IL-12 [22], and TNF-α [23] that determine chronic, ongoing, and continuous inflammation [24]. Various cytokines have been investigated affecting the immune cell profile in COPD. Also, an involvement of non-immune cells (e.g., epithelial cells, fibroblasts) in intercellular communication networks, as well as molecular mechanisms and their signalling patterns further influencing the pathological and clinical outcomes in COPD are especially important to mention. Overall, COPD is thought to be associated with various cytokines and their networks, moreover, increased expression of IL-1, IL-1 R, IL-4, IL-6, IL-7, IL-8, IL-8R, IL-12, TNF-α in COPD has been determined [25,26]. The association of Th1 inflammatory cytokines IL-1, IL-8, IL-12, and TNF-α, as well as Th2 inflammatory cytokines IL-6 and IL-10 with COPD has been stated [27]. 

Tissue remodelling processes are strongly affected by one of the most powerful and multifunctional molecules, transforming growth factor (TGF)-β1. Under the influence of TGF-β1, tissue morphologic changes do occur, including the widespread regulation of immune functions, inflammatory processes, tissue remodelling, fibrosis, and wound repair and healing. These multifunctional properties are variable, on the one hand, in an environment of other molecules (growth, inflammatory, anti-inflammatory, regulatory). On the other hand, TGF-β1 affects different tissue groups and cells [28]. In the determination of TGF-β1 effects, the common context of all ongoing changes in tissues must be respected [29]. In tissue damage caused by oxidative stress and inflammation reactions, extracellular matrix (ECM) degradation processes take place. Peptidases of tissue structural components (matrix metalloproteinases like matrix metalloproteinase (MMP)-2) degrade extracellular matrix collagen, elastin, basement membrane, laminin, fibronectin. Interestingly, degraded matrix components may further augment inflammatory processes [30]. Tissue inhibitor of matrix metalloproteinase (TIMP)-2 is a critical MMP-2 antagonist with a role to promote tissue damage healing and wound repair. TIMP-2 is then associated with better airway functional findings in COPD [31,32]. 

Antimicrobial peptides, including human β-defensins (hBD) like hBD-2, hBD-3, and hBD-4, are integral to innate host defence mechanisms. Various levels of hBD-2 expression have been found in the epithelia of different organs. Importantly, hBD-2 works synergistically together with other antimicrobial proteins, like lysozyme and lactoferrin [33], as well as it may work as a chemoattractant for monocytes, macrophages, neutrophils, and immature dendritic cells [34]. hBD-3 is thought to have bactericidal and antiviral functions [35]. hBD-3 is released in a continuous manner at basal levels or may be increased due to exact signalling, moreover, it also works as a chemoattractant [36]. hBD-4 has a strong antimicrobial nature, as well as higher functional activity when compared to hBD-2 and hBD-3 [37]. 

Numerous biomarkers from investigations of non-invasive samples have been evaluated in most of the COPD studies, whereas the local findings are limited due to the invasive nature of procedures to acquire airway biopsies and tissue material. Hereby, local findings within the airway wall provide valuable information and support the relevance of biomarkers in the pathogenesis of COPD. 

Thus, the aim of this study was to determine the appearance and relative distribution of various cytokines, chemokines, remodelling factors, regulatory factors, and antimicrobial peptides in COPD-affected lung tissue material in comparison with the normal control group.

## 2. Materials and Methods

### 2.1. Patients

In the patient group, COPD-affected lung tissue specimens were obtained from 40 patients (39 males and 1 female) aged from 53 to 88. Lung tissue material was obtained during flexible bronchoscopy of large airways by excision of a tissue specimen from the large bronchus wall. The diagnosis of stable COPD was assessed by clinical criteria and physical examination (e.g., assessment of symptoms, history of exposure to risk factors, objective findings) [38]. Additionally, spirometry and bronchoscopy findings were evaluated. 

In the control group, we evaluated lung tissue material obtained during a post mortem autopsy thoracotomy from 49 healthy control subjects (37 males and 12 females) aged 9 to 95 years by excision of a lung tissue fragment at the site of the large bronchus, including lung tissue parenchyma. Among the diagnoses of the control subjects, mostly unintentional major injuries due to trauma, self-harm, suicides, and sudden cardiac deaths were dated. 

All authors hereby declare that all study performances were examined and appropriately approved by the ethics committee and were therefore implemented in accordance with the ethical standards laid down in the 1964 Declaration of Helsinki. This study was independently reviewed and approved by the local Ethical Committee of Pauls Stradins Clinical University Hospital (Ethical code number: 230113-17 L, approval date: January 23, 2013), as well as written informed consent was obtained from all patients/legal representatives after the nature of the study had been fully explained.

### 2.2. Routine Histological Analysis

Approximately 1 cm^3^-sized specimens of lung tissue were obtained. Tissue material was fixed in 2% formaldehyde and 0.2% picric acid in 0.1 M phosphate buffer, then rinsed in Tyrode’s solution. The standard scheme of dehydration procedure was applied with 70% to 96% graded ethanol. Tissue material was cleansed using xylene. In the next procedure, tissue specimens were infiltrated using paraffin wax. Lung tissue sections were cut using a rotation microtome at 5 µm thickness. Sections were placed on the glass slides, then deparaffinised in xylene and rehydrated through graded series of 70% to 96% ethanol. Tissue sections were stained with hematoxylin and eosin [39,40]. 

### 2.3. Immunohistochemistry

Lung tissue sections were used to detect cytokines, chemokines, remodelling and regulatory factors, as well as antimicrobial peptides by using the biotin-streptavidin immunohistochemistry (IHC) method [41]. Tissue sections were deparaffinised with xylene and proceeded for a wash in ethanol, distilled water, and a wash buffer (tri-buffered saline; TRIS buffer). Sections were then placed in ethylenediaminetetraacetic acid (EDTA) boiling buffer. Lung tissue sections were cooled down and then washed twice in wash TRIS buffer, placed in 3% peroxide (H_2_O_2_), and then washed again in wash buffer. To dilute all antibodies, the chemical agent Antibody Diluent (ab64211; Abcam, Burlingame, CA, USA) was applied. Lung tissue specimens were incubated with HiDef DetectionTM reaction amplifier (code-954D-31, Sigma-Aldrich, Rocklin, CA, USA) for the detection of antibodies acquired from mouse or rabbit. Further washing in wash buffer (TRIS buffer) was performed. Incubation with HiDef DetectionTM horseradish peroxidase (HRP) polymer marker (code-954D-32, Sigma-Aldrich) was managed. ImmunoCruzTM avidin-biotin complex (ABC) staining system (sc-2018, Santa Cruz Biotechnology, Santa Cruz, CA, USA) was used for the detection of antibodies acquired from goat. Tissue sections were then incubated with blocking serum in TRIS buffer. Furthermore, lung tissue sections were incubated with secondary biotinylated goat antibody and the tertiary antibody after the application of wash buffer three times. These procedures were substantially followed by washing the tissue sections in a wash buffer and then processed with 3,3’-diaminobenzidine (DAB) Substrate Kit (code-957D-30; Sigma-Aldrich) to obtain immunoreactive structure staining in brown color. Afterwards, lung tissue sections were rinsed in distilled water and stained with haematoxylin within an appropriate procedure. In this study, we used mouse antibodies for the detection of IL-1α (sc-9983, diluted 1:50, Santa Cruz Biotechnology), IL-6 (sc-130326, 1:50, Santa Cruz Biotechnology), MMP-2 (sc-53630, 1:100, Santa Cruz Biotechnology), TIMP-2 (sc-21735, 1:100, Santa Cruz Biotechnology), Hsp-70 (585054A, 1:100, Invitrogen, Monza, Italy), hBD-4 (ab14419, 1:200, Abcam); rabbit antibodies for IL-4 (orb10908, 1:100, Biorbyt, Cambridge, UK), IL-7 (orb48420, 1:100, Biorbyt), IL-10 (P22301, 1:400, Nordic BioSite, Täby, Sweden), IL-12 (orb10894, 1:100, Biorbyt), TNF-α (ab6671, 1:100, Abcam), TGF-β1 (orb7087, 1:100, Biorbyt), hBD-2 (sc-20798, 1:200, Santa Cruz Biotechnology), hBD-3 (rb183268, 1:100, Biorbyt), and goat antibody for IL-8 (sc-1269, 1:50, Santa Cruz Biotechnology) by using the biotin-streptavidin immunohistochemistry method [41]. Lung tissue samples were examined by using bright field microscopy with a Leica DC 300F camera microscope (Leica DM500RB, Leica Biosystems Richmond, Richmond, VA, USA) for conventional histology and photography. Acquired images were analysed using Image-Pro Plus 6.0 software (Media Cybernetics, Silver Spring, MD, USA).

### 2.4. Quantification of Immunoreactive Cells

The appearance and local distribution of marker-containing immunoreactive cells was evaluated by a semi-quantitative grading method [42,43]. Immunoreactive cells were evaluated in following tissue and compartments of lung: bronchial epithelium, bronchial mucosal connective tissue, the wall of microcirculatory blood vessels in bronchial mucosa, bronchial smooth muscle, bronchial glands, bronchial cartilage, alveolar epithelium, alveolar macrophages. By counting the immunoreactive (positive) structures seen in the visual field, the following scale of the semi-quantitative method was used: 0 – no positive structures, 0/+ – occasional positive structures, + – few positive structures, +/++ – a few to a moderate number of positive structures, ++ – moderate number of positive structures, ++/+++ – moderate to numerous positive structures, +++ – numerous positive structures, +++/++++ – numerous to abundant positive structures, ++++ – an abundance of positive structures were observed in three random visual fields by magnification level X400 (ocular X10, objective X40) [43,44].

### 2.5. Data Statistical Analysis

We used non-parametric statistical methods to perform the statistical analysis. All the acquired data were ranked as ordinal values. The Mann–Whitney U Test [45] was conducted to determine a difference in the number of positive structures of each examined marker (cytokine, remodelling factor, or antimicrobial peptide) within exact lung tissue compartment in control and COPD groups. The statistical analysis was performed using the statistical program SPSS Statistics, version 23.0 (IBM Company, Chicago, IL, USA). Data of immunoreactive cell semi-quantitative count is presented as median values. In all the statistical analyses, two-tailed *p* values < 0.05 were considered statistically significant.

## 3. Results

### 3.1. Findings of Routine Histological Analysis

In COPD-affected lung tissue, various degrees of chronic inflammation and tissue remodelling in all examined COPD patients were evaluated by routine histological analysis with haematoxylin and eosin stain. Among the findings, goblet cell hyperplasia, squamous metaplasia of bronchial epithelium, basement membrane thickening, airway fibrosis, bronchial gland hypertrophy and hyperplasia, remodelling of bronchial microvasculature, smooth muscle cell hyperplasia and hypertrophy, and prominent inflammatory cell infiltration was noted. Dust-containing alveolar macrophages were found in almost all the COPD-affected lung tissue. Also, goblet cell hyperplasia of a large-calibre bronchus was evaluated with intermittent locations in respiratory epithelium. 

### 3.2. Findings of Immunohistochemistry

In the control group, the numbers of IL-8 immunoreactive cells were graded with values from no occasional (0/+) cells to moderate to numerous (++/+++) cells being the highest values (Table 1). 

Appearance and distribution of IL-4, IL-6, IL-12, and hBD-3 marked a range from occasional cells (0/+) to a few to moderate numbers (+/++) of immunoreactive cells. The numbers of IL-7 immunoreactive cells varied from no positive (0) to moderate (++) values. The numbers of IL-10, TIMP-2, and hBD-2 immunoreactive cells were evaluated from occasional (0/+) to moderate (++). The numbers of TNF-α, MMP-2, Hsp-70, and hBD-4 immunoreactive cells in the control group were evaluated with a range from no positive (0) cells to a few to moderate (+/++). The numbers of IL-1α and TGF-β1 positive cells varied from no positive cells (0) to few (+) being the lowest results. 

Overall, in the control group, the numbers of immunoreactive cells in bronchial epithelium, bronchial cartilage, alveolar epithelium, and among alveolar macrophages were evaluated mostly higher, whereas in bronchial mucosal connective tissue, blood vessel wall, smooth muscle, and glands they were mostly lower. We found higher numbers of immunoreactive cells positive for IL-4, IL-7, IL-10, and IL-12 in bronchial epithelium, IL-7, IL-8, IL-10, MMP-2, TIMP-2, Hsp-70, hBD-2, and hBD-3 in bronchial cartilage, TIMP-2, Hsp-70, hBD-2, hBD-3, and hBD-4 in alveolar epithelium, as well as IL-4, IL-6, IL-7, IL-8, IL-10, TNF-α, MMP-2, TIMP-2, and hBD-2 in alveolar macrophages.

**Figure 1:**
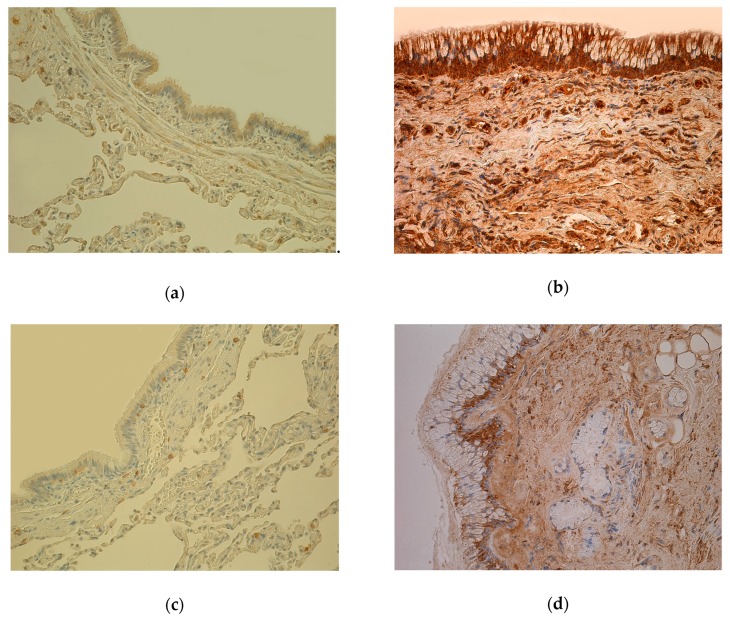

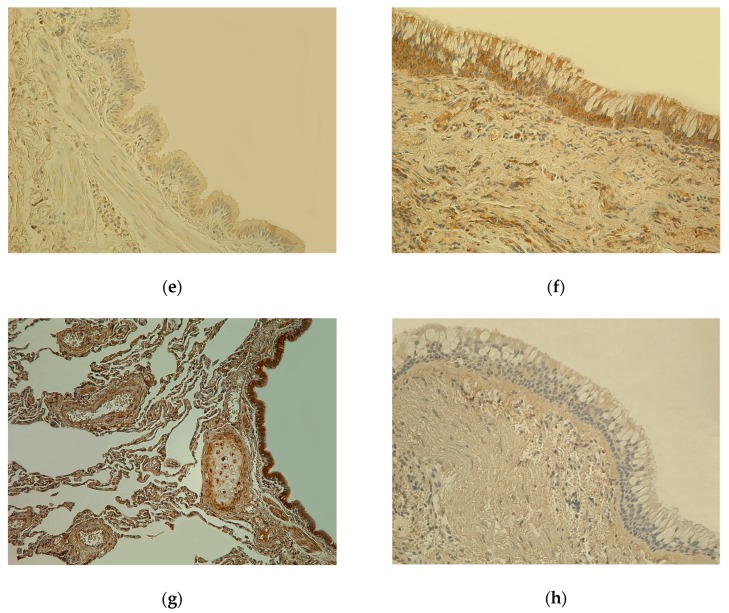
(**a**) Weakly stained few to moderate number (+/++) IL-7-positive cells in bronchial epithelium and smooth muscle; few (+) IL-7 positive cells in connective tissue of 81-year-old female bronchial wall (Control). IL-7 IHC, X200. (**b**) Numerous to abundance (+++/++++) of IL-7-containing cells in bronchial epithelium and connective tissue of 61-year-old male lung tissue (chronic obstructive pulmonary disease, COPD). IL-7 IHC, X200. (**c**) Almost no positive (0) IL-1α-containing cells in epithelium and few (+) IL-1α positive cells in connective tissue of 81-year-old female bronchial wall (Control). IL-1α IHC, X200. (**d**) Few to moderate number (+/++) IL-1α immunoreactive cells in epithelium and connective tissue of 78-year-old male bronchial wall (COPD). IL-1α IHC, X200. (**e**) Weakly stained few (+) hBD-2-containing cells in bronchial epithelium and connective tissue of 81-year-old female bronchial wall (Control). hBD-2 IHC, X200. (**f**) Moderate number (++) of hBD-2 immunoreactive cells in bronchial epithelium and connective tissue of 78-year-old male bronchial wall; goblet cell hyperplasia (COPD). hBD-2 IHC, X200. (**g**) Numerous (+++) Hsp-70-containing cells in bronchial epithelium and cartilage; moderate number (++) of Hsp-70-positive cells in connective tissue of 81-year-old female bronchial wall (Control). Hsp-70 IHC, X200. (**h**) Almost no positive (0) Hsp-70-containing cells in bronchial epithelium and connective tissue of 60-year-old male bronchial wall (COPD). Hsp-70 IHC, X200.

In the COPD group, we found a range of IL-7-containing cells from moderate numbers (++) to numerous amounts to an abundance (+++/++++) (Figure 1a,b). Moreover, overall these were the highest values of all examined markers (Table 2). 

The number of IL-1α (Figure 1b,c), MMP-2 immunoreactive cells varied from occasional positive cells (0/+) to moderate to numerous (++/+++) positive cells. IL-4, IL-8, TNF-α, TIMP-2 immunoreactive cells were evaluated in a range from few positive cells (+) to numerous (+++). In the COPD group, appearance and distribution of IL-6 immunoreactive cells were evaluated ranging from few (0/+) immunoreactive cells to moderate (++) numbers. In the COPD group, few to moderate numbers (+/++) to numerous (+++) immunoreactive cells for IL-10 were found. Numbers of IL-12, TGF-β1, hBD-2 (Figure 1e,f) immunoreactive cells in the COPD group were evaluated ranging from few (+) to moderate to numerous (++/+++). The findings of all cytokines and chemokines were more evident at inflammation sites with infiltrating immune cells visible and in granular tissue. The numbers of hBD-4 and hBD-4 immunoreactive cells ranged from no positive (0) and occasional (0/+) cells, to few to moderate numbers (+/++), respectively. The findings of Hsp-70 immunoreactive cells varied from no positive (0) cells to few (+) being the lowest result of all examined markers (Figure 1g,h). 

Mostly high numbers of IL-4, IL-7 (Figure 1c,d), IL-8, IL-10, IL-12 and TGF-β1 immunoreactive cells were evaluated among all examined tissues and bronchial compartments, whereas mostly low numbers were found of IL-6, Hsp-70 (Figure 1e,f) and hBD-4 immunoreactive cells. 

Overall, the highest numbers (from mostly moderate (++) to abundance (++++)) of IL-1α, IL-4, IL-7, IL-8, IL-10, IL-12, MMP-2, TIMP-2, TGF-β1 immunoreactive cells were marked increasingly in the blood vessel wall, connective tissue, and bronchial epithelium of COPD-affected lungs, respectively. Bronchial wall glands and smooth muscle were the compartments with the lowest numbers overall of all examined markers. 

In tissue material of three COPD patients stained for ten markers, bronchial cartilage was found. It presented markedly high numbers (from few (+) to numerous (+++)) of immunoreactive cells of IL-1α, IL-4, IL-6, TNF-α, MMP-2, TIMP-2, Hsp-70, hBD-2, hBD-3, hBD-4. For examined markers IL-1α, TNF-α, Hsp-70, hBD-2, hBD-4, bronchial cartilage was the bronchial wall location with the highest evaluation of immunoreactive cells out of all examined compartments. 

### 3.3. Findings of Data Statistical Analysis

With some exceptions, the Mann–Whitney U Test determined statistically significant (*p* < 0.05) higher numbers of IL-1α, IL-4, IL-6, IL-7, IL-8, IL-10, IL-12, TNF-α, MMP-2, TIMP-2, TGF-β1, hBD-2 immunoreactive cells in bronchial epithelium, connective tissue, blood vessel wall, bronchial smooth muscle, and bronchial glands of COPD-affected lungs in comparison with the control group. There was no statistically significant (*p* < 0.05) difference between the numbers of IL-6 immunoreactive cells in bronchial epithelium (U = 616, Z = –1.284, *p* = 0.199), IL-8 immunoreactive cells in bronchial blood vessel wall (U = 849.5, Z = –0.625, *p* = 0.532), hBD-3 immunoreactive cells in bronchial smooth muscle (U = 778.5, Z = –0.411, *p* = 0.681) and bronchial glands (U = 509, Z = –1.483, *p* = 0.138), as well as hBD-4 immunoreactive cells in bronchial epithelium (U = 769.5, Z = –0.51, *p* = 0.61), connective tissue (U = 871.5, Z = –0.612, *p* = 0.541) of COPD-affected lungs in comparison with the control group. The Mann–Whitney U Test determined statistically significant (*p* < 0.05) lower numbers of Hsp-70 in bronchial epithelium, connective tissue, blood vessel wall, bronchial smooth muscle, and bronchial glands of COPD-affected lungs in comparison with the control group.

## 4. Discussion

In control group lung tissue, we found evidence of moderately increased numbers of immunoreactive cells for all examined markers, indicating basal levels of various mediators (cytokines, remodelling factors, antimicrobial substances) released at relative health status. In bronchial epithelium, bronchial cartilage, alveolar epithelium, and among the alveolar macrophages, we found the highest numbers of immunoreactive cells of control group subjects. Moreover, we could identify more pronounced findings of immunoreactive cells in bronchial epithelium, in bronchial cartilage, in alveolar epithelium, as well as among alveolar macrophages. Various cells and their communication with other tissue structures design and shape signalling pathways to form local immunity in lung at relative health status [46]. Immunity concepts have been reviewed with questions considering the possible non-immune cell response to various inflammation-associated mediators, as well as the production of their own signalling mediators. As the firstly exposed surface, the epithelium actively regulates local immunity. Due to continuous antigen exposure, activated epithelial cells might recruit locally inhabiting immunocompetent cells (e.g., macrophages). Epithelial cells use autocrine and paracrine signalling pathways to provide intercellular communication [47]. Lung tissue immunity holds necessary structures and forces for pathogen recognition, also maintaining various immune response types and general tissue repair after cell and tissue damage. Overall, the lung innate and adaptive immune system in its steady state generally maintains an anti-inflammatory environment [48]. Failure of immune response regulation might easily lead to a ruined normal lung tissue architecture and structure due to mostly molecular structural changes [49]. 

In COPD-affected lungs, we found more pronounced findings of IL-4, IL-7, IL-8, IL-10, IL-12 and TGF-β1 immunoreactive cells among all examined tissues and bronchial compartments, whereas mostly low numbers were found of IL-6, Hsp-70 and hBD-4 immunoreactive cells. Numerous studies explain COPD association with various cytokine gene and their networks, as well as with increased local expression of cytokines IL-1, IL-4, IL-6, IL-7, IL-8, IL-12, and TNF-α [26,27]. COPD is associated with Th1 inflammatory cytokines IL-1, IL-8, IL-12, and TNF-α, as well as Th2 inflammatory cytokines IL-6 and IL-10 [28]. 

The highest numbers of IL-1α, IL-4, IL-7, IL-8, IL-10, IL-12, MMP-2, TIMP-2, TGF-β1 immunoreactive cells were marked increasingly in the blood vessel wall, connective tissue, and bronchial epithelium of COPD-affected lungs, respectively. Bronchial wall glands and smooth muscle were the compartments with the lowest numbers of immunoreactive cells positive for all examined markers. Continuous gaseous exposures may affect the release of various cytokines within airway epithelial cells. COPD phenotype of airway epithelial cells able to release various cytokines has been reported [50]. Airway epithelial cells are activated by and further release various cytokines (e.g., IL-1α, IL-4, IL-6, IL-8, and IL-10) [46,47]. Furthermore, epithelial cells are capable to produce inflammatory, anti-inflammatory, and regulatory mediators as IL-1, IL-6, IL-8, TNF-α in a response to different stimuli [9,51]. 

Statistically significant (*p* < 0.05) higher numbers of immunoreactive cells positive for all examined markers, but not for Hsp-70 and hBD-4, were found in COPD-affected airways when compared to the control group. This finding indicates activated numerous signalling pathways in COPD, as well as promotes the local significance of these markers in COPD pathogenesis [10,26]. IL-1α may be associated with a first-time initiation, as well as further maintenance and amplification of complex inflammatory responses in COPD with more highlighted essentials of airway epithelium as a primary release site. Moreover, release of IL-1α from immune and non-immune cells can initiate a whole cascade of other cytokine release [52]. IL-4 signalling is associated with COPD pathogenesis, where IL-4 effects of mucus hypersecretion [53], mucus gland hyperplasia, smooth muscle cell hypertrophy, and hyperplasia do occur [54]. We may suggest IL-4 is associated with mentioned histopathological findings in routine histological findings of COPD. Since IL-6 is a pleiotropic cytokine with multiple functions and possibly may switch controversial anti- and pro-inflammatory functions due to the local signalling environment [14], we may suggest IL-6 is associated with an equilibrium of inflammatory and anti-inflammatory signalling in COPD [55]. Although TNF-α is one of the most studied cytokines in COPD research, it was not the dominant inflammatory cytokine in our study. TNF-α induces the release of other pro-inflammatory cytokines (e.g., IL-8) having a wide range of pro-inflammatory properties [15,56]. Increased IL-12 supports its role in inflammatory processes, and moreover, it confirms COPD over asthma findings in our examined airway tissue [57,58,59,60]. More pronounced findings of IL-8 in our study supports the leading role of IL-8 in inflammation associated with COPD. Cytokine IL-8 typically participates in neutrophilic inflammation [61]. One of the major sources of inflammatory cytokine IL-8 is lung airway epithelium [15,50], that promotes the increased findings of numerous (+++) IL-8 immunoreactive cells in airway epithelium presented by our study. Possibly, IL-8 may be the dominant inflammatory cytokine in COPD. Due to the stimulation with cytokines (e.g., IL-6, IL-8, IL-12, TNF-α), various immune cells participate in ongoing inflammatory responses with further remodelling of the airway wall [62,63]. 

Out of all examined markers, IL-7 immunoreactive cell numbers were the greatest. Highly increased IL-7 findings may propose its supreme regulatory role [64]. 

High numbers of IL-10 immunoreactive cells were marked in COPD-affected airways. IL-10 is a potent anti-inflammatory cytokine, moreover, the release of IL-10 maintain a suppressive effect on inflammatory processes [65]. IL-10 balances inflammation and tissue damage by following the pattern of inflammatory cytokine immunoreactivity in all examined tissues and compartments. Hereby, the findings of increased anti-inflammatory cytokine IL-10 in our study point to its protective role. 

In COPD, air-derived irritants might harm epithelial tissue on-site, moreover, these harms can further initiate inflammation with attracting immune cells and activating proteolytic environment with the predominance of collagenase MMP-2 [10]. Overall the number of TIMP-2 immunoreactive cells was greater than the number of MMP-2 immunoreactive cells. This may point to TIMP-2’s protective and regulatory role in remodelling since TIMP-2 directly inhibits MMP-2 to maintain stability of ECM environment [66]. From the aspect of COPD-affected tissue, TGF-β1 release is uniquely based on oxidant-mediated mechanisms confirming the activating role of oxidative stress. Airway epithelium, smooth muscle, fibroblasts, and myofibroblasts are important sources of TGF-β1 in the lung, moreover, TGF-β1 is orchestrating tissue remodelling [32]. TGF-β1 exhibits a protective role by struggling to hold lung tissue homeostasis in physiological conditions that are usually lost in COPD [67]. 

Low numbers of Hsp-70 immunoreactive cells estimated in our study might occur if prolonged stress stimuli hold cellular and tissue stress responses. Although Hsp-70 holds the protective role against cytotoxic damage and it is up-regulated under various physical and chemical stress stimuli, substantial adaption to chronic oxidative stress occurs through down-regulation of Hsp-70 [68,69].

Increased numbers of hBD-2 and hBD-3, but not hBD-4, may suggest their association with COPD morphopathogenesis. Both hBD-2 and hBD-3 are participating in innate host defense system against possible bacterial colonization characteristically found along with COPD [35,36]. 

Interestingly, bronchial cartilage held higher numbers of immunoreactive cells positive for IL-7, IL-8, IL-10, MMP-2, TIMP-2, Hsp-70, hBD-2, and hBD-3 in control group tissue. In three COPD-affected patients, comparatively high numbers of immunoreactive cells were found in bronchial cartilage. Hyaline cartilage of lung airways has been poorly analysed for both health and disease status. We may speculate the pronounced cytokine and antimicrobial peptide distribution in bronchial cartilage suggests the involvement of a compensatory local immune response in the supporting tissue. Findings of remodelling markers in cartilage may suggest the potential of hyaline cartilage plasticity in health and disease.

## 5. Conclusions

In healthy lung tissue, the appearance and distribution of factors involved in local immunity, remodelling and antimicrobial defence suggest persistent, continuous, and possibly adapting low-level expressions, indicating the presence of local regulatory and modulating patterns at relative health status.

Increased numbers of IL-1α, IL-4, IL-6, IL-7, IL-8, IL-10, IL-12, and TNF-α immunoreactive cells suggest the extensive presence and local significance of these markers in COPD pathogenesis. The dominance of immunoreactive cells in the COPD-affected airway epithelium over other tissue compartments highlights the essentials of epithelium in inflammatory and defence signalling with more pronounced findings of IL-1α, IL-4, IL-6, IL-7, IL-8, IL-10, MMP-2, TIMP-2, TGF-β1, hBD-3. Wide distribution and extensive appearance of increased MMP-2 and TGF-β1 but decreased Hsp-70 proposes pronounced tissue damage and remodelling in COPD, particularly being suppressed by TIMP-2. High numbers of hBD-2 and hBD-3 immunoreactive cells may highlight these markers in COPD to maintain antimicrobial activity within stable regulation of local immunity.

## Figures and Tables

**Table 1:** Immunoreactivity of cytokines, chemokines, remodelling factors, regulatory factors, and antimicrobial peptides in the control group by semi-quantitative grading. Summary of median values and interquartile ranges.

KERRYPNX	Control (N = 49)							
	Bronchial Epithelium	Bronchial Mucosal Connective Tissue	Bronchial Mucosal Blood Vessel Wall	Bronchial Smooth Muscle	Bronchial Glands	Bronchial Cartilage	Bronchial Epithelium	Bronchial Mucosal Connective Tissue
	Mdn (Q_2_) IQR							
**IL-1α**	0/+	0/+	0	0	0	+	0/+	+
	1.0	1.0	0.5	0	0	0.5	1.0	1.0
**IL-4**	+/++	0/+	0/+	0	0/+	0/+	+	+/++
	1.5	0.5	1.0	1.0	1.0	1.0	1.25	1.5
**IL-6**	+	0/+	0/+	0	0	+	+	+/++
	1.5	0.5	1.0	0.5	0.5	1.5	0.5	0.5
**IL-7**	+/++	0/+	+	0	0/+	++	+	+/++
	1.0	1.0	0.75	1.0	1.0	1.0	1.25	1.5
**IL-8**	0/+	+	++	0/+	+	++/+++	+	++
	0.5	1.0	2.0	0.5	1.0	0.5	1.0	1.5
**IL-10**	+/++	+	+	0/+	0/+	++	+	+/++
	1.0	0.5	1.0	0.5	1.0	1.0	1.5	1.0
**IL-12**	+/++	0/+	+	+/++	+/++	+	+	0/+
	1.0	0.5	0.5	1.0	0.5	1.0	0.5	0.5
**TNF-α**	0	0/+	0	0	0	0/+	0/+	+/++
	0.5	1.0	0.5	0.5	0.5	0.5	0.5	1.5
**MMP-2**	+	0/+	0/+	0	0/+	+/++	+	+/++
	1.0	0.5	1.5	1.0	0.5	1.5	1.0	1.0
**TIMP-2**	+	0/+	+	0/+	0/+	++	+/++	+/++
	1.0	0.5	1.0	0.5	0	1.0	1.0	1.0
**TGF-β1**	+	0/+	0/+	0	0/+	+	+	+
	1.0	0.25	1.0	0.5	1.0	0.5	1.0	1.25
**Hsp-70**	+	0/+	0/+	0	0/+	+/++	+/++	+
	0.5	0.5	1.0	0.5	0.5	0.5	0.5	1.0
**hBD-2**	0/+	0/+	+	0	0/+	++	+/++	+/++
	1.0	1.0	2.0	0.5	0.5	1.5	0.5	1.5
**hBD-3**	0/+	0/+	0/+	0/+	0/+	+/++	+/++	+
	0.5	0.5	1.5	1.0	1.0	1.0	1.0	2.0
**hBD-4**	0/+	0/+	0/+	0	0	+	+/++	+
	0.75	1.0	1.5	0.5	1.0	1.5	0.5	2.5

Semi-quantitative grading scores are displayed with rank values. “Control”– Control group, “N”–Number of the study subjects, “Mdn”– Median value, “IQR”– interquartile range (with 95% CI–95% Confidence interval), “Q_2_”–2nd Quartile (50th percentile value).

**Table 2:** Immunoreactivity of cytokines, chemokines, remodelling factors, regulatory factors, and antimicrobial peptides in the COPD group by semi-quantitative grading. Summary of median values and interquartile ranges.

	COPD (N = 40)					
	Bronchial Epithelium	Bronchial Mucosal Connective Tissue	Bronchial Mucosal Blood Vessel Wall	Bronchial Smooth Muscle	Bronchial Glands	Bronchial Cartilage
	Mdn (Q_2_) IQR					
**IL-1α**	++/+++	+/++	+	0/+	+	++/+++
	1.5	1.5	1.5	0.5	1.0	
**IL-4**	+++	++	++	+	+/++	++
	1.5	1.5	1.5	1.0	1.0	
**IL-6**	++	0/+	+	0/+	0/+	+
	2.0	1.0	1.0	0	1.0	
**IL-7**	+++/++++	++/+++	++/+++	+/++	++	
	1.5	1.5	1.5	1.5	2.0	
**IL-8**	+++	++	++	+	+/++	
	2.0	1.0	1.5	1.0	1.75	
**IL-10**	+++	++/+++	++/+++	+/++	+/++	
	2.0	1.5	1.5	1.5	2.0	
**IL-12**	++/+++	++/+++	++	+	+	
	1.5	1.0	1.5	0.5	0.5	
**TNF-α**	++	++	++	+	+	+++
	1.0	1.5	1.5	0.5	1.5	
**MMP-2**	++/+++	++	+/++	0/+	+	++
	1.5	2.0	1.5	1.0	1.0	
**TIMP-2**	+++	++	+/++	+/++	+	++
	2.0	1.5	1.5	2.0	1.5	
**TGF-β1**	++/+++	++/+++	++/+++	+/++	+	
	1.5	1.5	1.5	1.5	1.5	
**Hsp-70**	0	0	0	0	0	+
	0.5	0.5	2.0	0	0.5	
**hBD-2**	+/++	++	++	+	+	++/+++
	1.5	2.0	1.5	1.0	0.5	
**hBD-3**	++	++	+/++	0/+	+	+/++
	1.5	1.0	1.0	0.5	0.5	
**hBD-4**	0/+	0/+	0/+	0	0	+/++
	1.0	1.0	0.5	0	0.25	

Semi-quantitative grading scores are displayed with rank values. “Control”– Control group, “N”–Number of the study subjects, “Mdn”– Median value, “IQR”– interquartile range (with 95% CI–95% Confidence interval), “Q_2_”– 2nd Quartile (50th percentile value).
